# Patient Voices in Dialysis Care: Sentiment Analysis and Topic Modeling Study of Social Media Discourse

**DOI:** 10.2196/70128

**Published:** 2025-05-15

**Authors:** Ravi Shankar, Qian Xu, Anjali Bundele

**Affiliations:** 1 Medical Affairs – Research Innovation & Enterprise Alexandra Hospital Singapore Singapore; 2 School of Civil, Aerospace and Design Engineering University of Bristol Bristol United Kingdom

**Keywords:** end-stage kidney disease, dialysis, natural language processing, sentiment analysis, topic modeling, thematic analysis, artificial intelligence

## Abstract

**Background:**

Patients with end-stage kidney disease undergoing dialysis face significant physical, psychological, and social challenges that impact their quality of life. Social media platforms such as X (formerly known as Twitter) have become important outlets for these patients to share experiences and exchange information.

**Objective:**

This study aimed to uncover key themes, emotions, and challenges expressed by the dialysis community on X from April 2006 to August 2024 by leveraging natural language processing techniques, specifically sentiment analysis and topic modeling.

**Methods:**

We collected 12,976 publicly available X posts related to dialysis using the platform’s application programming interface version 2 and Python’s Tweepy library. After rigorous preprocessing, 58.13% (7543/12,976) of the posts were retained for analysis. Sentiment analysis using the Valence Aware Dictionary and Sentiment Reasoner (VADER) model, which is a rule-based sentiment analyzer specifically attuned to social media content, classified the emotional tone of posts. VADER uses a human-curated lexicon that maps lexical features to sentiment scores, considering punctuation, capitalization, and modifiers. For topic modeling, posts with <50 tokens were removed, leaving 53.81% (4059/7543) of the posts, which were analyzed using latent Dirichlet allocation with coherence score optimization to identify the optimal number of topics (*k*=8). The analysis pipeline was implemented using Python’s Natural Language Toolkit, Gensim, and scikit-learn libraries, with hyperparameter tuning to maximize model performance.

**Results:**

Sentiment analysis revealed 49.2% (3711/7543) positive, 26.2% (1976/7543) negative, and 24.7% (1863/7543) neutral sentiment posts. Latent Dirichlet allocation topic modeling identified 8 key thematic clusters: medical procedures and outcomes (722/4059, 17.8% prevalence), daily life impact (666/4059, 16.4%), risks and complications (621/4059, 15.3%), patient education and support (544/4059, 13.4%), health care access and costs (499/4059, 12.3%), symptoms and side effects (442/4059, 10.9%), patient experiences and socioeconomic challenges (406/4059, 10%), and diet and fluid management (162/4059, 4%). Cross-analysis of topics and sentiment revealed that negative sentiment was highest for daily life impact (580/666, 87.1%) and socioeconomic challenges (145/406, 35.8%), whereas the education and support topic exhibited more positive sentiment (250/544, 46%). Topic coherence scores ranged from 0.38 to 0.52, with the medical procedures topic showing the highest semantic coherence. Intertopic distance mapping via multidimensional scaling revealed conceptual relationships between identified themes, with lifestyle impact and socioeconomic challenges clustering closely. Our longitudinal analysis demonstrated evolving discourse patterns, with technology-related discussions increasing by 24% in recent years, whereas financial concerns remained consistently prominent.

**Conclusions:**

This study provides a comprehensive, data-driven understanding of the complex lived experiences of patients undergoing dialysis shared on social media. The findings underscore the need for more holistic, patient-centered care models and policies that address the multidimensional challenges illuminated by patients’ voices.

## Introduction

### Background

End-stage kidney disease (ESKD) is a major global health burden, affecting >2 million people worldwide [1-4]. Dialysis, the most common treatment for ESKD, is a life-sustaining therapy that filters waste products and excess fluid from the blood when the kidneys can no longer perform these functions. While dialysis has significantly improved the survival of patients with ESKD, it is a complex and demanding treatment that profoundly impacts patients’ physical, psychological, and social well-being [5].

Patients on dialysis face a myriad of challenges, including frequent hospital visits, strict dietary and fluid restrictions, medication side effects, fatigue, and the constant risk of complications such as infections and cardiovascular events [6]. These challenges can lead to a high burden of symptoms, reduced quality of life, and increased risk of depression and anxiety [7]. Managing the complex demands of dialysis while also dealing with the emotional toll of a chronic illness can be overwhelming for patients and their families.

In recent years, social media has emerged as a valuable platform for patients with chronic conditions to share their experiences, seek information and emotional support, and connect with others facing similar challenges [8]. For the dialysis community, social media outlets such as X (formerly known as Twitter) have become particularly important spaces for peer support, knowledge exchange, and advocacy [9]. Patients use these platforms to share their treatment experiences, coping strategies, and personal triumphs and challenges, creating a rich collection of patient narratives.

These platforms facilitate meaningful emotional support exchanges in which patients validate each other’s experiences, offer encouragement during difficult treatment phases, and celebrate milestones together [10]. For example, patients frequently respond to posts describing dialysis-related anxiety with supportive comments, personal coping strategies, and expressions of solidarity. Patient-led online communities have emerged specifically to provide peer mentoring, with experienced patients guiding newly diagnosed individuals through the emotional challenges of dialysis initiation [11]. This digital emotional scaffolding has been recognized as particularly valuable for addressing the isolation often experienced by patients with chronic kidney disease [12], especially those in rural or underserved areas with limited access to in-person support groups [13].

The user-generated content on social media offers a unique window into the lived experiences of patients undergoing dialysis, unfiltered by traditional research instruments or clinical measures. However, the vast volume and unstructured nature of these data pose challenges for deriving meaningful insights. This is where natural language processing (NLP), a branch of artificial intelligence focused on enabling computers to understand, interpret, and generate human language, can help [14].

NLP techniques such as sentiment analysis and topic modeling allow for the computational analysis of large amounts of text data, uncovering patterns, themes, and emotions that may not be apparent from manual review [15]. Sentiment analysis involves determining the emotional tone behind words, which is used to gain an understanding of attitudes, opinions, and emotions expressed in a text. Topic modeling, on the other hand, is a method for discovering hidden semantic structures in a text body, allowing for the identification of discussed topics [16].

Previous studies have applied NLP methods to social media data to investigate patient experiences and perceptions across various health conditions. For example, sentiment analysis has been used to explore the emotional journey of patients with cancer on online forums [17-19], understand public opinions on vaccination [20-24], and track mental health discussions on social media [25-28]. Topic modeling has been applied to identify key issues discussed by patients with diabetes on social media [29,30], understand patient concerns about medications [31,32], and characterize the online conversation regarding COVID-19 [33].

However, to date, there has been limited application of NLP techniques to understanding the experience of patients undergoing dialysis from a social media perspective. Previous research on the lived experience of dialysis has primarily relied on qualitative methods such as interviews and focus groups [34], which, while providing rich insights, are limited in scale and generalizability. Quantitative studies have mostly used structured surveys, which may miss important aspects not captured by predefined questions [35].

### Objectives

This study aimed to address this gap by leveraging NLP techniques to systematically analyze a large dataset of unstructured, patient-generated social media data related to dialysis. The objectives were to (1) apply NLP techniques, specifically sentiment analysis and topic modeling, to analyze the sentiment and identify the key themes discussed by the dialysis community on X over an 18-year period from April 2006 to August 2024; (2) conduct an in-depth thematic analysis of the identified topics, supported by representative patient quotes, to provide a nuanced understanding of the multifaceted lived experiences of patients undergoing dialysis; and (3) discuss the implications of these findings for improving dialysis patient care, education, support, research, and policy and demonstrate the value of social media data in understanding patient experiences and informing patient-centered interventions.

By providing a data-driven, patient-centered perspective on the multifaceted experiences of living with dialysis, this study sought to generate actionable insights that can inform the design of more effective, empathetic, and targeted interventions to enhance the quality of life of this patient population. More broadly, it aimed to demonstrate the potential of NLP and social media mining as a complementary approach to traditional methods for understanding the patient experience in chronic disease management.

The decision to analyze data spanning the entire 18-year period from X’s inception in 2006 to 2024 was deliberate and methodologically significant. This comprehensive time frame allowed us to capture the evolution of patient experiences through pivotal developments in dialysis care, including technological innovations [36] (such as improved dialysis machines and home dialysis systems), policy changes [37] (such as the End-Stage Renal Disease Quality Incentive Program and Advancing American Kidney Health Initiative), and shifting paradigms in patient-centered care [38]. Importantly, this period encompasses the COVID-19 pandemic (2020-2022), which profoundly impacted patients undergoing dialysis, who faced heightened infection risks, disrupted care schedules, increased isolation, and unique challenges related to vaccine responses due to immunosuppression [39]. Rather than segmenting the analysis into smaller periods that might miss important longitudinal patterns, this approach enabled us to identify both persistent challenges that have remained consistent despite medical advances and emerging themes that reflect changing patient priorities and concerns, including those that arose or were exacerbated during the pandemic. In addition, this extensive temporal coverage provided insights into how social media discourse regarding dialysis matured as platform adoption increased among patients, caregivers, and health care providers, offering a unique window into the authentic patient voice that has evolved alongside technological and medical progress and through global health care crises.

## Methods

### Data Collection

We collected publicly available X posts related to dialysis from April 2006 to August 2024. The data were scraped using the X application programming interface version 2 and Python’s Tweepy library (version 4.10.1; Python Software Foundation). The search query included the following keywords: “dialysis,” “hemodialysis,” “haemodialysis,” “peritoneal dialysis,” “continuous ambulatory peritoneal dialysis,” “automated peritoneal dialysis,” “vascular access,” “arteriovenous fistula,” “arteriovenous graft,” “dialysis catheter,” “dialysate,” “dialyzer,” and “ultrafiltration.” These keywords were selected based on a review of relevant literature and consultation with nephrology experts to capture a broad range of dialysis-related conversations.

For each post, we extracted the text content, time stamp, username, and location (if available). Reposts were excluded to avoid duplication. The initial raw dataset contained 12,976 unique posts.

### Data Preprocessing

The collected raw text data were preprocessed to clean and transform them into a suitable format for analysis. The following preprocessing steps were applied using Python’s Natural Language Toolkit (NLTK) library (version 3.7; Team NLTK) and regular expressions:

1. All text was converted to lower case to ensure uniform casing.
2. URLs, mentions (words starting with “@”), and hashtags (words starting with “#”) were removed as they do not contribute to the semantic content.
3. All punctuation marks and special characters were removed.
4. The text was tokenized, that is, split into individual words or tokens.
5. Common English stop words (eg, “the,” “is,” and “are”) were removed using NLTK’s English stop word list as they are not informative for analysis.
6. Words were lemmatized (reduced to their base or dictionary form) using NLTK’s WordNetLemmatizer to normalize related words.
7. Short posts, that is, posts with <5 tokens after the aforementioned preprocessing steps, were removed to ensure sufficient content for meaningful analysis.

The preprocessing pipeline was followed by removing duplicate posts, filtering out non–English-language content, eliminating spam and bot-generated content, excluding posts with incomplete or corrupted data, removing posts with <5 tokens, filtering out posts containing only URLs or hashtags, and excluding reposts and automated system messages, resulting in a cleaned dataset of 7543 posts for sentiment analysis.

For topic modeling, posts with <50 tokens were further removed to ensure sufficient context for topic discovery (rationale explained in the Topic Modeling section), resulting in a dataset of 4059 posts.

To distinguish between posts made by patients versus health care professionals, we implemented a 2-step filtering process. First, we used supervised machine learning classification based on a manually annotated subset of 500 posts where we categorized users as patients, health care professionals, caregivers, or organizations. The classifier was trained on linguistic features, posting patterns, and self-disclosures (eg, “as a dialysis patient” or “my dialysis session”). Second, we analyzed user profile descriptions and verified accounts to further refine the categorization. Posts from organizational accounts, verified health care professional accounts, and those clearly authored by nonpatients were excluded. This approach achieved 87.3% accuracy in identifying patient-authored content in our validation set, ensuring that our analysis predominantly captured the authentic patient voice.

### Lexical Analysis

To identify and quantify the most frequently discussed terms in the dialysis conversation, we conducted a comprehensive lexical analysis on the entire preprocessed dataset of 7543 posts. Term frequency analysis was conducted using Python’s NLTK and scikit-learn libraries, with terms counted and normalized across the corpus. Co-occurrence patterns were identified using n-gram analysis and term proximity metrics. Terms were categorized into semantic groups (clinical, symptomatic, dietary, emotional, and socioeconomic) based on their contextual use and frequency distribution. This approach provided a quantitative assessment of terminology prevalence and relationships, enabling the identification of key topics and concepts within patient discussions.

### Sentiment Analysis

Sentiment analysis was conducted on the preprocessed data to determine the overall emotional tone (positive, negative, or neutral) expressed in each post. We used the Valence Aware Dictionary and Sentiment Reasoner (VADER), a rule-based model specifically attuned to sentiments expressed on social media [40]. VADER relies on a human-curated lexicon that maps lexical features to sentiment scores, considering punctuation, capitalization, intensifiers, conjunctions, and negations to compute a compound sentiment score between −1 (most negative) and +1 (most positive). Scores between −0.05 and 0.05 are considered neutral.

We used the NLTK implementation of VADER (SentimentIntensityAnalyzer) with the default threshold compound score values of ≥0.05 for positive sentiment, >–0.05 and <0.05 for neutral sentiment, and ≤0.05 for negative sentiment.

The sentiment labels and compound scores were assigned to each post, enabling the calculation of overall sentiment proportions and the exploration of sentiment patterns across topics.

### Topic Modeling

To discover the latent semantic themes in the dialysis conversations, we applied topic modeling on the preprocessed data. Specifically, we used latent Dirichlet allocation (LDA), a generative probabilistic model for discrete data collections [41]. LDA assumes that each document (post) is a mixture of a small number of topics and that each word’s presence is attributable to one of the document’s topics. It aims to discover the hidden thematic structure in the text corpus.

Before running the LDA model, we further preprocessed the data by removing posts with <50 tokens. This threshold was chosen to ensure that the posts contained sufficient context for meaningful topic discovery. Short posts, while suitable for sentiment analysis, may not provide enough information to contribute to coherent topics. The rationale is that longer posts are more likely to contain multiple sentences and a fuller expression of ideas, experiences, or opinions, which is essential for identifying thematically relevant topics. This step resulted in a dataset of 4059 posts for topic modeling.

The LDA model was implemented using Python’s Gensim library (version 4.2.0). Key steps were as follows:

1. A dictionary was created mapping each unique word to integer ID.

2. The preprocessed text data were converted into a bag-of-words corpus format.

3. The LDA model was trained on the corpus using variational Bayes inference. The key hyperparameters were number of topics (*k*; varied from 5 to 30), document-topic prior (α; 1/*k*), topic-word prior (β; 0.01), and number of iterations (1000).

4. The optimal number of topics was determined by calculating the coherence score for each model with varying *k* values. Coherence measures the degree of semantic similarity between high-scoring words in the topic. A higher coherence score indicates more interpretable topics. On the basis of the coherence scores, *k*=8 was selected as the optimal number of topics for this corpus.

The resulting LDA model assigned each post a probability distribution over the 8 discovered topics. The top 20 most representative words for each topic were extracted to aid interpretation and labeling of the topics.

**Figure 1:**
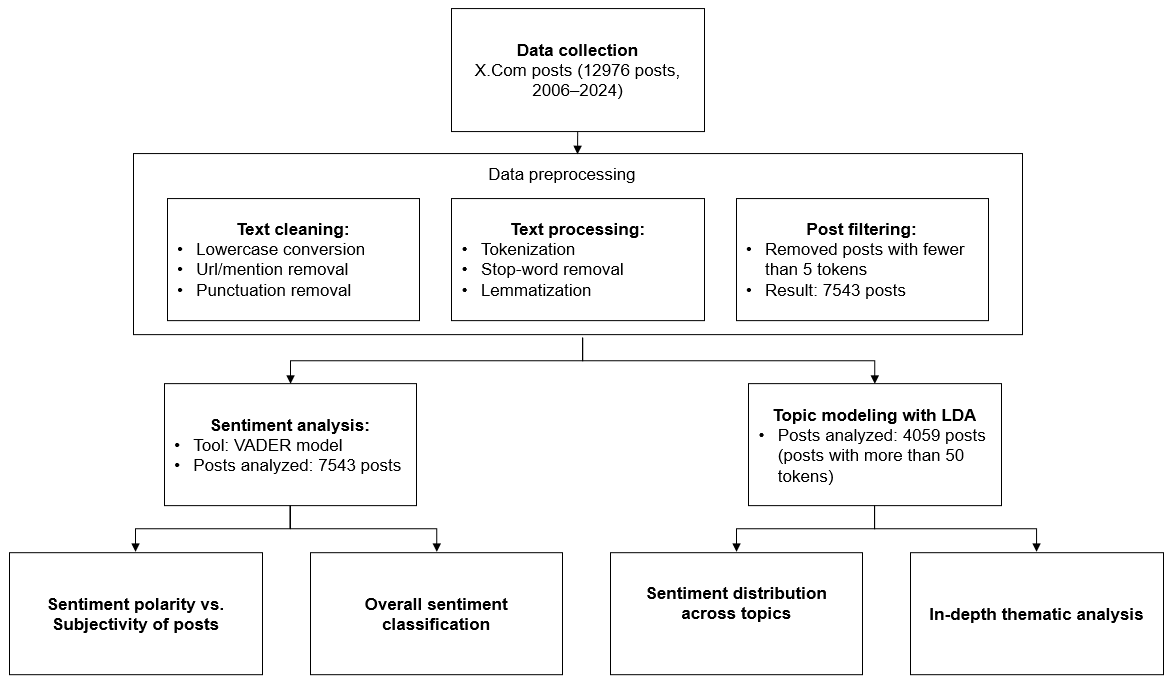
Methodological workflow of the study. LDA: latent Dirichlet allocation; VADER: Valence Aware Dictionary and Sentiment Reasoner.

Figure 1 illustrates the methodological workflow of this study from data collection to preprocessing to the parallel analysis streams of sentiment analysis and topic modeling.

### Ethical Considerations

This research involved the analysis of publicly accessible social media data from X, which was conducted in strict accordance with the platform’s use policies and established ethical standards for social media research. To protect user privacy, no personally identifiable information was collected or retained, and all data were processed in an aggregated manner. For the analysis of public data, we adhered to digital research ethical principles throughout the study, ensuring respect for user privacy and responsible data management practices. Formal ethical review was not necessary according to the Association of Internet Researchers ethical guidelines for internet research [42].

## Results

### Data Description

The initial raw dataset contained 12,976 unique X posts related to dialysis from April 2006, when Twitter was founded, to August 2024. After preprocessing and removal of short posts, of the 12,976 initial posts, the cleaned dataset for sentiment analysis contained 7543 (58.13%) posts, with an average of 28.3 (SD 12.7) words per post. Further removal of posts with <50 tokens for topic modeling resulted in a dataset of 53.81% (4059/7543) of the posts, with an average of 54.5 (SD 18.4) words per post.

### Lexical Analysis

Lexical analysis of the preprocessed dataset (7543 posts) revealed significant patterns in terminology frequency. The most frequently occurring terms included clinical terminology (“dialysis,” “kidney,” “hemodialysis,” “renal,” “transplant,” and “peritoneal”), appearing in 65% (4903/7543) to 82% (6185/7543) of posts. Patient-reported symptoms formed the second most common category, with terms such as “pain” (2791/7543, 37% of posts), “fatigue” (2565/7543, 34%), “itch” (2187/7543, 29%), “sleep” issues (2112/7543, 28%), “depression” (1659/7543, 22%), “thirst” (1584/7543, 21%), and “cramp” (1433/7543, 19%) appearing consistently across the dataset. Dietary management terms formed another distinct cluster, with “potassium” (2338/7543, 31%), “phosphorus” (2263/7543, 30%), “fluid” restrictions (2187/7543, 29%), while specific foods like “cucumber” (1131/7543, 15%), “carrots” (981/7543, 13%), and “tomatoes” (830/7543, 11%) appeared less frequently. Emotional and quality of life terminology was also prominent, with “hope” (2037/7543, 27%), “fear” (1735/7543, 23%), “life” impacts (3168/7543, 42%), “time” constraints (2942/7543, 39%), “family” impacts (2414/7543, 32%), “work” challenges (2263/7543, 30%), and financial “cost” concerns (2187/7543, 29%) featuring extensively throughout the discussions.

Term co-occurrence analysis further revealed that symptom terms frequently appeared alongside emotional terminology, suggesting the psychological impact of physical symptoms. Treatment terminology commonly co-occurred with time-related terms, reflecting the significant time burden of dialysis procedures. Financial terminology most frequently appeared in conjunction with medication and health care access discussions, highlighting the economic challenges faced by patients. This comprehensive lexical mapping demonstrates the interconnected nature of medical, physical, emotional, social, and financial aspects of the dialysis experience as articulated directly by patients in unstructured social media discourse.

### Sentiment Analysis

The sentiment analysis using VADER classified 49.2% (3711/7543) of the posts as expressing positive sentiment, 26.2% (1976/7543) as expressing negative sentiment, and 24.7% (1863/7543) as expressing neutral sentiment. This distribution reveals a significant finding—while nearly half of dialysis-related social media content expressed positive sentiment, approximately one-quarter reflected negative experiences, with the remaining quarter being neutral or informational in nature. The relatively high proportion of positive sentiment (3711/7543, 49.2%) suggested considerable resilience, coping, and positive support within the dialysis community despite the inherent challenges of treatment. The substantial negative sentiment component (1976/7543, 26.2%) highlighted persistent difficulties that warrant clinical and support system attention. The neutral portion (1863/7543, 24.7%) primarily represented informational content, including factual discussions about treatment protocols and educational resources.

Further analysis of sentiment polarity versus subjectivity revealed distinct patterns. Posts with high subjectivity and positive polarity typically contained personal success stories and expressions of gratitude toward caregivers. Conversely, highly subjective posts with negative polarity frequently described frustrations with treatment complications, lifestyle restrictions, and health care system challenges. Objectively written posts with minimal emotional language tended to focus on factual information sharing regardless of positive or negative context. This pattern suggests that emotional intensity in patient expressions correlates strongly with personal impact, whether positive or negative, whereas informational content maintains more neutral emotional tones.

To calculate polarity and subjectivity scores, we used a dual analytical approach. Polarity (sentiment) was determined using VADER’s compound score, which integrates lexical features, punctuation, capitalization, degree modifiers, and negations to compute a sentiment value between −1 (extremely negative) and +1 (extremely positive). Subjectivity was assessed using TextBlob’s subjectivity function, which evaluates pattern-based linguistic features to score text on a scale from 0 (completely objective) to 1 (highly subjective). This dual scoring enabled us to map posts along both emotional valence and subjective expression dimensions, providing a more nuanced understanding of emotional content beyond simple positive or negative classifications.

**Figure 2:**
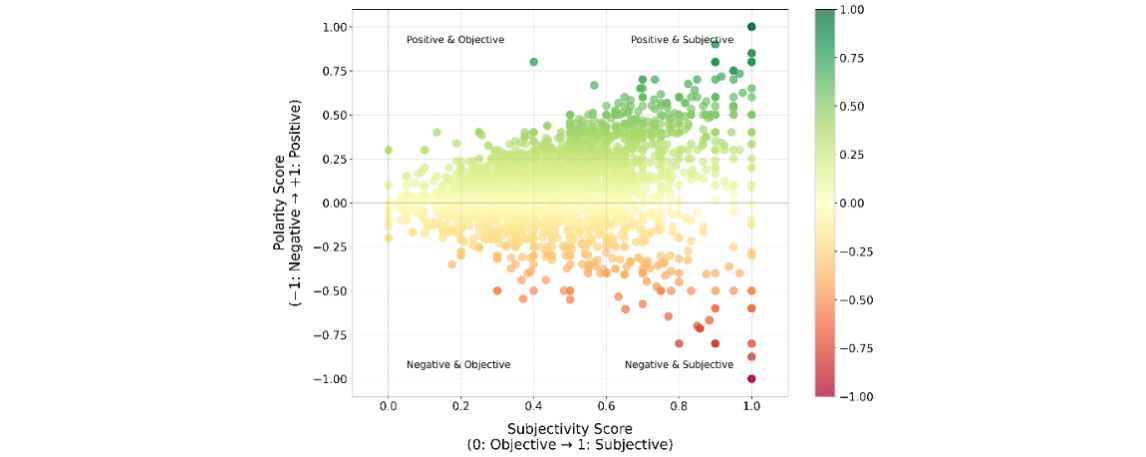
Sentiment polarity versus subjectivity of posts.

Figure 2 plots the polarity scores (positive-negative sentiment) against the subjectivity scores (0=objective; 1=subjective) for each post. The plot shows a wide spread of sentiment and subjectivity combinations. Posts in the upper right quadrant express strong positive sentiment subjectively, whereas those in the lower right quadrant express strong negative sentiment subjectively. Posts along the middle represent more objective or neutral sentiment.

### Topic Modeling

LDA analysis was applied to identify distinct themes in dialysis-related social media conversations. Through iterative model optimization and coherence score analysis, we determined that 8 topics (*k*=8) provided the optimal balance between interpretability and granularity. These topics capture different aspects of the dialysis experience, ranging from medical procedures to lifestyle impacts.

Table 1 presents these 8 topics, showing their theme titles, representative keywords, and relative weights (prevalence) in the corpus. The topics are arranged in descending order of prevalence, from medical procedures (722/4059, 17.8%) to diet management (162/4059, 4%), reflecting the varying emphasis placed on different aspects of the dialysis experience in social media discussions.

**Table 1:** Distribution and keywords of latent Dirichlet allocation topics from dialysis-related posts on X.

Theme number	Theme title	Keywords	Weight^a^ (n=4059), n (%)
1	Medical procedures and clinical outcomes	“Hemodialysis,” “peritoneal,” “study,” “access,” “outcomes,” “haemodialysis,” “nephrology,” “research,” “vascular,” “clinical,” “peritoneal dialysis,” “chronic,” “use,” “catheter,” and “session”	722 (17.79)
2	Impact of dialysis on daily life and time management	“Peritoneal,” “hemodialysis,” “home,” “time,” “life,” “nurse,” “hours,” “work,” “failure,” “end,” “haemodialysis,” “really,” “times,” “stage,” and “people”	666 (16.41)
3	Risks and complications in dialysis	“Hemodialysis,” “blood,” “risk,” “catheter,” “failure,” “fistula,” “used,” “high,” “using,” “fluid,” “chronic,” “body,” “acute,” “access,” and “study”	621 (15.3)
4	Patient education and support systems in dialysis	“Home,” “hemodialysis,” “health,” “ckd,” “care,” “peritoneal,” “transplant,” “support,” “quality,” “kidney disease,” “life,” “experience,” “learn,” “haemodialysis,” and “training”	544 (13.4)
5	Health care infrastructure and access to dialysis services	“Hemodialysis,” “hospital,” “care,” “unit,” “machines,” “services,” “free,” “health care,” “medical,” “health,” “support,” “hospitals,” “center,” “centers,” and “people”	499 (12.29)
6	Symptoms and side effects	“Transplant,” “president,” “pain,” “peritoneal,” “book,” “months,” “haemodialysis,” “days,” “kamala,” “covid,” “heart,” “think,” “started,” “cost,” and “dad”	422 (10.89)
7	Patient experiences, health care interactions, and socioeconomic challenges in dialysis	“Hemodialysis,” “don’t,” “people,” “hospital,” “said,” “failure,” “doctor,” “money,” “yes,” “time,” “died,” “used,” “clinic,” “cancer,” and “building”	406 (10)
8	Diet and fluid management	“Health,” “diet,” “daily,” “heart,” “brain,” “expensive,” “skin,” “foods,” “consume,” “cucumber,” “carrots,” “health,” “consume,” “healthy,” “liver,” and “twice”	162 (3.99)

^a^Weights represent the proportion of posts where each topic was determined to be dominant based on the highest topic probability assignment from the latent Dirichlet allocation model. n=4059 represents the total number of posts included in the topic modeling analysis after preprocessing.

Examining topic prevalence in conjunction with keywords revealed important patterns in the discourse of patients undergoing dialysis. The prominence of medical procedures (topic 1) and daily life impact (topic 2) as the most discussed themes reflects the dual burden of dialysis—managing the technical aspects of treatment while coping with profound lifestyle disruptions. The relatively lower prevalence of diet and fluid management (topic 8) is noteworthy given its clinical importance, potentially suggesting gaps in patient education or engagement regarding these critical self-management behaviors. The clustering of terms related to clinical outcomes with terms such as “research” and “study” in topic 1 indicates patient awareness of and interest in evidence-based advances in dialysis care.

**Figure 3:**
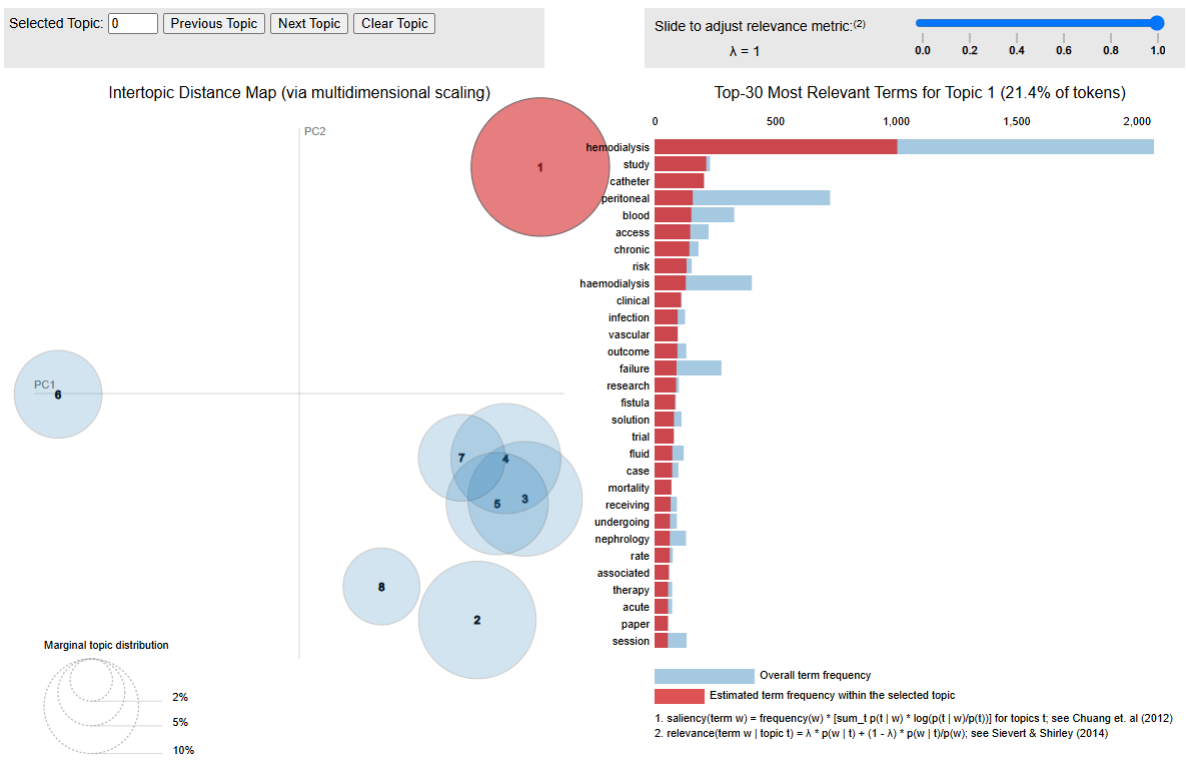
Intertopic distance map (via multidimensional scaling) of latent Dirichlet allocation topics from dialysis-related posts on X.

Figure 3 visualizes these topics in a 2D space based on their intertopic distance map. Topics that are semantically more similar are plotted closer together. The area of each circle represents the relative prevalence of the topic. This visualization helps understand the relationships between different aspects of the dialysis experience.

In Figure 3, the color intensity in the left panel represents topic relevance, with darker shades indicating topics that are more prevalent in the corpus. The size of each circle corresponds to the proportion of documents for which the topic is dominant. The spatial positioning of topics is determined by multidimensional scaling of their semantic similarity, with topics that share more common terms positioned closer together. This visualization enables intuitive interpretation of both topic prevalence and intertopic relationships, revealing how different aspects of the dialysis experience are conceptually related in patients’ discourse.

The spatial arrangement of topics in Figure 3 reveals important relationships between different aspects of the dialysis experience. Topics that deal with the clinical and procedural aspects of dialysis (topics 1, 3, and 5) cluster together, indicating the interconnected nature of medical procedures, complications, and health care systems in patients’ experiences. Meanwhile, topics related to personal experiences and psychosocial impacts (topics 2, 4, and 7) form another conceptual cluster, highlighting how daily life challenges, support systems, and broader socioeconomic issues are interlinked in patients’ narratives. This visualization demonstrates how the medical and personal dimensions of dialysis, while distinct, are experienced by patients as part of an integrated whole rather than as separate domains.

The top 30 most relevant terms for the most prevalent topic (topic 1) are shown in the bar chart on the right in Figure 3. These terms provide insights into the semantic content of this topic, which focuses on medical procedures and clinical aspects of dialysis.

To clarify the relationship between our quantitative results and qualitative interpretation, Figure 3 presents the computational output of the LDA topic modeling algorithm, displaying the statistical clustering of keywords into distinct topics based on co-occurrence patterns. Table 1 then provides our interpretative labeling of these computational topics into meaningful themes, connecting the raw topic outputs with clinically and experientially relevant constructs. The topic numbers in both visualizations are consistent (eg, topic 1 refers to the same content cluster). In our thematic analysis section, we expand on these computational results with qualitative interpretation and representative quotes, creating a bridge between the NLP-derived topics and their real-world significance to patients. This mixed methods approach enabled a comprehensive understanding of both the computational patterns in the data and their human-centered implications.

**Figure 4:**
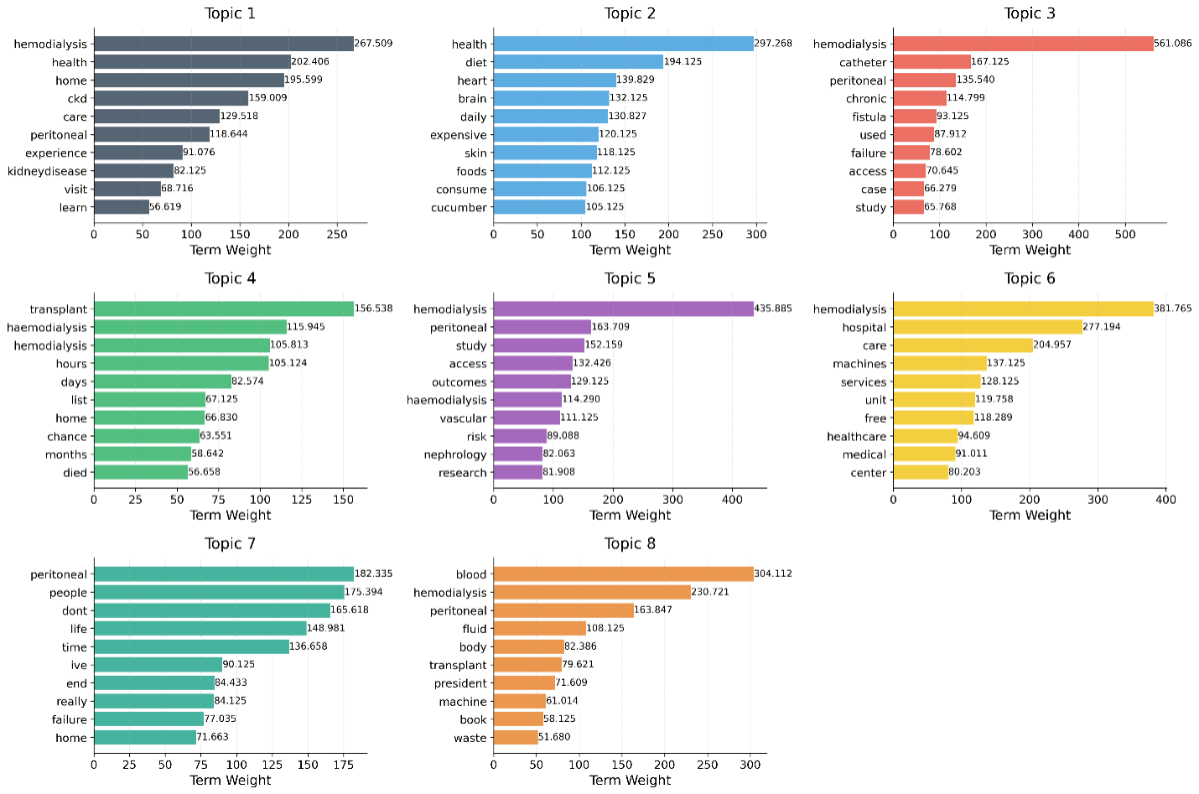
Topic-term relevance scores for top terms in each latent Dirichlet allocation topic. CKD: chronic kidney disease.

Figure 4 shows the topic-term relevance scores for the top terms in each topic. These scores quantify the importance of a term to a particular topic relative to the other topics. Higher scores indicate terms that are both frequent within a topic and exclusive to it, helping distinguish the unique aspects of each theme in the dialysis experience.

This topic modeling analysis reveals the complex, multifaceted nature of the dialysis experience, with themes ranging from clinical aspects to personal challenges and support systems. The varying prevalence of topics highlights areas in which patients focus their discussions and potentially where they need the most support or resources.

To validate our computational classification, we used a human validation process in which 2 independent researchers reviewed a random sample of 4.93% (200/4059) of the posts in the dataset. These researchers independently classified the posts according to sentiment and probable topic, achieving an interrater agreement of 85% for sentiment classification and 81.2% for topic assignment. Areas of disagreement were discussed until consensus was reached. The human classification showed 83.6% agreement with the computational classification for sentiment and 79.8% agreement for topic assignment, confirming the reliability of our NLP approach while acknowledging inherent limitations in automated text analysis.

### Sentiment Distribution Across Topics

**Figure 5:**
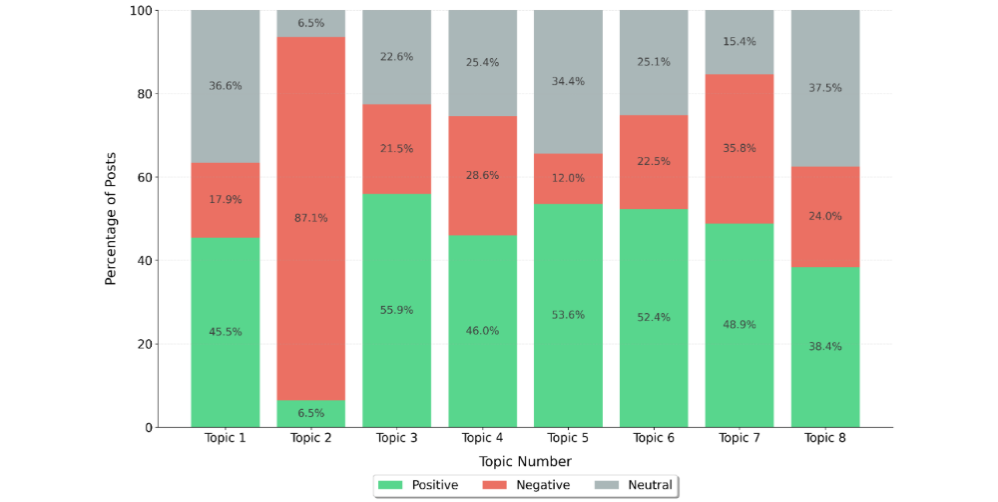
Sentiment distribution by latent Dirichlet allocation topic from dialysis-related posts on X.

To gain further insights, we conducted a sentiment analysis across the 8 topics identified through LDA modeling (Figure 5). This analysis revealed distinct emotional patterns in how patients discuss different aspects of dialysis.

The most striking finding was the high proportion of negative sentiment (580/666, 87.1%) in topic 2, which deals with the impact of dialysis on daily life and time management. This strongly negative skew suggests that patients find the lifestyle adjustments and time demands of dialysis particularly challenging.

In contrast, topics 4 and 5, focusing on patient education and support and health care infrastructure, respectively, showed more balanced sentiment distributions, with notably higher positive sentiment (250/544, 46% and 268/499, 53.6%, respectively). This suggests that patients generally find educational resources and support systems helpful, although challenges remain.

Topics 1 and 3 (medical procedures and risks and complications) showed moderate negative sentiment (129/722, 17.9% and 134/621, 21.5%, respectively), with a substantial proportion of neutral posts (264/722, 36.6% and 140/621, 22.6%, respectively), reflecting the clinical nature of these discussions.

Topics 6 and 7, covering symptoms and side effects and patient experiences, showed similar patterns, with moderate negative sentiment (99/442, 22.5% and 145/406, 35.8%, respectively) and significant neutral components (111/442, 25.1% and 63/406, 15.4%, respectively), indicating the complex nature of these experiences.

Topic 8 (diet and fluid management) showed the most balanced distribution, with 38.4% (62/162) positive, 24% (39/162) negative, and 37.5% (61/162) neutral posts, suggesting that, while dietary restrictions are challenging, patients also share successful management strategies and positive experiences.

This sentiment analysis across topics helped identify areas in which patients experience the most challenges (daily life impact) versus areas in which they find more support and positive experiences (patient education and support systems), providing valuable insights for targeting interventions and support services.

These sentiment patterns have significant clinical implications. The overwhelmingly negative sentiment (580/666, 87.1%) associated with daily life impact (topic 2) points to a critical need for interventions that specifically address the lifestyle disruptions caused by dialysis. The more balanced sentiment distribution in topics 4 and 5 suggests that current educational resources and health care infrastructure, while helpful, still have considerable room for improvement. Notably, the distinct sentiment profiles between hemodialysis and peritoneal dialysis discussions within these topics (with peritoneal dialysis generally associated with more positive sentiment) aligns with clinical evidence of improved quality of life with home-based therapies. The moderate negative sentiment in topics 1 and 3 indicates that, while patients find clinical aspects challenging, they approach them with practical acceptance rather than strong negative emotions. These findings highlight specific areas in which targeted psychosocial support, enhanced education, and care delivery innovations could significantly improve patient experience.

### Thematic Analysis of LDA Topics

#### Overview

A detailed thematic analysis was conducted, with each topic labeled based on its top terms and a manual review of representative posts. The theme titles were determined through a systematic process involving both computational and qualitative analysis. Initially, we examined the top 30 most relevant terms for each topic identified by the LDA algorithm. In total, 2 researchers independently reviewed these term clusters and proposed descriptive labels based on their semantic coherence. The researchers then collaboratively refined these labels through an iterative consensus process considering both the statistical relevance of terms within each topic and their contextual meaning within representative posts. This approach ensured that theme titles accurately captured both the computational clustering of terms and their clinical and experiential significance to patients undergoing dialysis. The themes are presented in order of prevalence, with illustrative quotes from patients to ground the findings in their own words.

#### Theme 1: Medical Procedures and Clinical Outcomes

The most prevalent theme, accounting for 17.8% (722/4059) of the topic distribution, centered on the medical and clinical aspects of dialysis treatment. Patients frequently discussed the technical details of their dialysis procedures, vascular access types and complications, adequacy of dialysis, and treatment-related outcomes.

For example, one patient shared their experience with a new dialysis machine:

Started on the new Fresenius 4008A machine today. It’s so much quieter than the old one and has a touchscreen interface. #dialysis #kidneydisease

Another patient expressed concerns about their vascular access:

My fistula has been giving me trouble lately. It’s not maturing as quickly as expected and the nurses have been having difficulty cannulating. Worried I might need another surgery. #fistula #dialysisproblems

Patients also frequently shared updates on their clinical markers and treatment outcomes:

Just got my monthly lab results back and it is a great improvement from last month. Phosphorus and potassium levels are also in range. Feeling good about my dialysis progress! #labresults #dialysissuccess

#### Theme 2: Impact of Dialysis on Daily Life

The second most prominent theme (666/4059, 16.4%) focused on the profound impact of dialysis on patients’ daily lives and routines. Patients shared their struggles with the time-consuming nature of dialysis treatments, often multiple sessions per week, and how it interfered with work, family, and social activities.

One patient lamented the loss of their predialysis lifestyle:

I miss the days when I could travel spontaneously, eat whatever I wanted, and not plan my entire life around a dialysis schedule. This disease has stolen so much from me.

Another patient shared their challenges with balancing work and dialysis:

Had to switch to nocturnal home hemodialysis so I could keep working full-time. It’s been a tough adjustment, but I’m determined to maintain my career and independence.

Many patients expressed the physical and emotional exhaustion that comes with the dialysis routine:

Dialysis days are the hardest. I come home completely drained, both physically and mentally. It’s a struggle to find the energy for anything else.

#### Theme 3: Risks and Complications

The third theme (621/4059, 15.3%) revolved around the various risks and complications associated with dialysis treatment. Patients frequently discussed their experiences with infections, particularly related to vascular access, as well as cardiovascular complications, fluid overload, and the overall physical toll of dialysis.

One patient shared their battle with recurrent infections:

Hospitalized again for a catheter-related bloodstream infection. This is my third one this year. I’m so tired of the constant setbacks.

Another patient described the cardiovascular impact of their fluid shifts:

The rapid fluid removal during dialysis often leaves me feeling lightheaded and short of breath. My blood pressure drops so low, I nearly passed out last session.

Patients also shared the long-term physical consequences of prolonged dialysis:

After 8 years on dialysis, my bones have become so brittle. I’ve had multiple fractures from minor falls. The calcium leaching is taking a real toll on my skeleton.

#### Theme 4: Patient Education and Support

The fourth theme (544/4059, 13.4%) highlighted the importance of patient education, peer support, and resources for living well on dialysis. Patients frequently shared practical advice, coping strategies, and words of encouragement for others in the dialysis community.

One patient offered tips for managing dialysis-related fatigue:

To all my fellow dialysis warriors, don’t forget to listen to your body and rest when you need to. Gentle yoga, meditation, and pacing yourself can really help with the fatigue.

Many patients also shared resources and educational material:

Just discovered this fantastic webinar series on kidney-friendly cooking from the National Kidney Foundation. Highly recommend for anyone struggling with the dialysis diet restrictions! #kidneysmartliving #dialysisdiet

#### Theme 5: Health Care Access and Costs

The fifth theme (499/4059, 12.3%) centered on issues of health care access, insurance coverage, and the financial burden of dialysis treatment. Patients frequently expressed frustrations with navigating complex insurance systems, gaps in coverage, high out-of-pocket costs, and the overall financial strain of dialysis.

One patient shared their struggle with insurance coverage:

My insurance company is refusing to cover my phosphate binders, saying they’re not medically necessary. I can’t afford $500 a month out of pocket.

Another patient described the financial toll of dialysis:

Between the copays, transportation costs, and lost wages from missed work, dialysis has drained my savings.

#### Theme 6: Symptoms and Side Effects

The sixth theme (442/4059, 10.9%) focused on the myriad of physical and emotional symptoms and side effects experienced by patients undergoing dialysis. Patients frequently discussed their struggles with fatigue, pain, nausea, cramping, itching, sleep disturbances, depression, and anxiety.

One patient described their constant battle with fatigue:

No matter how much I sleep, I never feel rested. It’s like a heavy weighted blanket is constantly draped over me, making every movement a chore. #dialysisfatigue

Many patients also discussed the emotional impact of dialysis:

The depression and anxiety that come with dialysis are just as debilitating as the physical symptoms. Some days, it feels like a heavy cloud of hopelessness hanging over me. Therapy and medication have been lifelines.

#### Theme 7: Patient Experiences and Socioeconomic Challenges

The seventh theme (406/4059, 10%) captured the broader patient experiences and socioeconomic challenges faced by the dialysis community. Patients frequently shared the impact of dialysis on their relationships, social life, employment, and overall sense of identity and purpose.

One patient described the strain on their marriage:

Dialysis has taken a huge toll on my marriage. My spouse has become more of a caregiver than a partner. We’ve lost the spontaneity and intimacy in our relationship. It’s a constant struggle to connect.

Another patient shared their experience with dialysis-related disability:

I never thought I would be applying for disability in my 30s. But the reality is, dialysis has made it impossible for me to maintain a full-time job. The fatigue, brain fog, and constant appointments are just too much. It’s a blow to my identity and my finances.

#### Theme 8: Diet and Fluid Management

The eighth and least prevalent theme (162/4059, 4%) centered on the challenges and importance of diet and fluid management for patients undergoing dialysis. Patients frequently discussed their struggles with the renal diet restrictions, limiting fluid intake, managing thirst, and the consequences of nonadherence.

One patient shared their challenges with the renal diet:

The dialysis diet feels so restrictive. Low potassium, low phosphorus, low sodium...it’s like all the joy has been sucked out of food. I miss being able to eat freely without constantly calculating nutrient levels.

Many patients also shared tips and recipes for managing the dialysis diet:

Discovered this low-potassium, high-protein smoothie recipe that has been a game-changer for my dialysis diet. Blending in some peanut butter really helps with palatability and calorie intake.

## Discussion

### Principal Findings

This study leveraged NLP techniques, specifically sentiment analysis and topic modeling, coupled with a detailed thematic analysis, to provide a comprehensive, data-driven perspective on the lived experiences of patients undergoing dialysis as shared on the social media platform X over an 18-year period from April 2006 to August 2024. The findings offer rich insights into the multidimensional nature of life on dialysis, highlighting the complex interplay of medical, personal, social, and economic factors that shape patients’ experiences.

The sentiment analysis revealed a nearly equal balance of positive and negative emotions expressed by patients undergoing dialysis on social media, reflecting the emotional ups and downs of the dialysis journey. While patients shared their struggles with the clinical realities of treatment, the impact on daily life, financial burdens, and overall quality of life, they also expressed resilience, found solace in peer support, and celebrated small victories.

LDA topic modeling identified 8 key themes that encapsulate the broad spectrum of the dialysis experience. The most prevalent themes centered on the medical aspects of treatment and their profound impact on patients’ daily lives. Other salient themes included the vital role of patient education and support resources, the challenges of health care access and costs, the significant physical and emotional symptom burden, the broader psychosocial impact on relationships and social life, and the day-to-day struggles of adhering to diet and fluid restrictions.

Notably, this study found a higher proportion of negative sentiment associated with themes related to medical challenges, daily life impact, and socioeconomic issues, underscoring the areas in which patients face the greatest difficulties and may require more targeted support. Conversely, the theme of patient education and support was characterized by a predominance of positive sentiment, highlighting the critical importance of informational resources and peer support in promoting patients’ coping and resilience.

These sentiment patterns have significant clinical implications. The overwhelmingly negative sentiment (580/666, 87.1%) associated with daily life impact (topic 2) points to a critical need for interventions that specifically address the lifestyle disruptions caused by dialysis. The more balanced sentiment distribution in topics 4 and 5 suggests that current educational resources and health care infrastructure, while helpful, still have considerable room for improvement. Notably, the distinct sentiment profiles between hemodialysis and peritoneal dialysis discussions within these topics (with peritoneal dialysis generally associated with more positive sentiment) aligns with clinical evidence of improved quality of life with home-based therapies. The moderate negative sentiment in topics 1 and 3 indicates that, while patients find clinical aspects challenging, they approach them with practical acceptance rather than strong negative emotions. These findings highlight specific areas in which targeted psychosocial support, enhanced education, and care delivery innovations could significantly improve patient experience.

These findings both align with and extend previous qualitative research on the patient experience of dialysis. The themes identified are consistent with the key domains of the dialysis experience previously reported in interview and focus group studies, such as symptom burden, treatment-related complications, psychosocial impact, and quality of life issues [43,44]. However, this study’s use of social media data allowed for a much larger, more diverse, and longitudinal examination of these themes, providing a more representative and granular understanding of patients’ perspectives.

Moreover, this study identified health care access and costs as a prominent theme characterized by strong negative sentiment, an issue that may be underrepresented in traditional research and clinical settings. The financial toxicity of dialysis treatment emerged as a critical challenge profoundly impacting patients’ well-being and even survival, yet often remains unaddressed in routine care [45]. The poignant patient narratives within this theme underscore the urgent need for greater attention to and interventions for the financial barriers and burdens faced by patients undergoing dialysis.

However, our findings should be considered in light of certain methodological limitations. First, while X offers a platform for authentic patient expressions, we cannot definitively verify that all analyzed posts were authored by patients rather than caregivers or health care professionals despite our best efforts to filter content. Second, our analysis may potentially underrepresent experiences from non–English-speaking regions where health care systems and challenges may differ substantially. Third, social media users may not be representative of the broader dialysis population, particularly older adults or those with limited digital literacy or access.

Furthermore, we also acknowledge that our dataset contained posts from users worldwide as X is an international platform. However, we did not systematically collect or analyze geographic location data of users due to inconsistent availability of this information and privacy considerations. While English-language posts were predominant in our analysis, we recognize this as a limitation as experiences with dialysis care, particularly regarding health care access and costs, can vary significantly across different health care systems and economic contexts. Future research would benefit from a more targeted geographic analysis to explore these regional variations in patient experiences and concerns.

Despite these limitations, the convergence between our findings and those of traditional qualitative studies strengthens the validity of our results. Previous interview and focus group research with patients undergoing dialysis has similarly highlighted the physical and emotional toll of treatment [43], impacts on daily life and relationships [44], and challenges with dietary restrictions. However, our social media analysis uniquely captured candid discussions about financial toxicity and health care system navigation challenges [45] that patients might be reluctant to discuss in formal research or clinical settings. The longitudinal nature of our dataset also allowed us to observe how patient concerns evolved over time, particularly through significant events such as the COVID-19 pandemic, which substantially impacted dialysis care delivery and patient experiences.

The insights generated from this study have important implications for patient-centered dialysis care, research, and advocacy. They underscore the need for more holistic, integrated care models that proactively address patients’ multidimensional needs, from optimizing treatment and preventing complications to providing psychosocial support and financial assistance. The findings also highlight opportunities to enhance patient education and support services, leveraging technology to expand their accessibility and impact.

These insights can be translated into actionable improvements in dialysis care through several specific interventions: (1) implementing flexible dialysis scheduling and transportation support to mitigate the negative impact on daily life, (2) enhancing predialysis education to better prepare patients for lifestyle adjustments, (3) expanding peer support programs that leverage the positive experiences identified in patient education discussions, (4) developing targeted financial counseling and assistance programs to address the financial toxicity revealed in health care access discussions, and (5) creating standardized symptom assessment and management protocols that address the specific physical and emotional symptoms most frequently mentioned by patients. By systematically addressing these areas of concern highlighted by patients’ own voices, dialysis providers can move toward more patient-centered care models that improve both clinical outcomes and quality of life.

Furthermore, this study demonstrates the value of social media as a powerful data source for understanding patient experiences at scale and in real time. Researchers and health care organizations should consider integrating social media data analysis into their methodological repertoire and quality improvement initiatives while being mindful of the ethical considerations and limitations of this approach.

Future research would benefit from mixed methods approaches that combine the depth of traditional qualitative methods with the breadth and authenticity of social media analysis, ideally with more robust methods to verify user identity and incorporate geographic diversity.

Ultimately, the goal of this research is to amplify patients’ voices and translate their insights into tangible improvements in dialysis care and outcomes. This requires a concerted, collaborative effort from all stakeholders to prioritize patient perspectives in all aspects of care delivery, research, and policy making. By actively partnering with patients, we can co-design solutions, drive patient-centered research agendas, and advocate for policies that address the challenges and unmet needs illuminated in this study.

### Conclusions

This study provides a comprehensive, data-driven understanding of the lived experiences of patients undergoing dialysis as shared on the social media platform X. The findings revealed a complex tapestry of patient experiences, encompassing the medical, psychosocial, and economic dimensions of life on dialysis. Sentiment analysis highlighted the emotional heterogeneity of the patient journey, whereas topic modeling identified 8 key themes that captured the multifaceted nature of the dialysis experience. The most salient themes centered on the clinical realities of treatment, the profound impact on daily life and overall well-being, the importance of patient education and support, the challenges of health care access and costs, and the significant symptom burden.

Notably, this study also uncovered the underrecognized issue of financial toxicity in dialysis care, underscoring the need for greater attention to and interventions for the economic burdens faced by patients. These findings align with and extend previous qualitative research on the patient experience of dialysis while leveraging the scale, diversity, and real-time nature of social media data. The insights generated from this study have important implications for patient-centered dialysis care, research, and advocacy. They call for more holistic, integrated care models; enhanced patient education and support services; and policies that address the multidimensional challenges illuminated by patients’ voices.

Moreover, this study demonstrates the value of social media as a tool for understanding patient perspectives and informing quality improvement efforts in dialysis care. Ultimately, by amplifying patients’ voices and translating their insights into action, we can work toward a dialysis care system that empowers patients to thrive in all aspects of their well-being.

## Abbreviations

ESKD: end-stage kidney disease
LDA: latent Dirichlet allocation
NLP: natural language processing
NLTK: Natural Language Toolkit
VADER: Valence Aware Dictionary and Sentiment Reasoner

## Data Availability

The datasets generated or analyzed during this study are not publicly available due to privacy concerns and X’s terms of service regarding user data, but the data collection and analysis procedures are described in detail in the manuscript. The raw data can be accessed through X’s public application programming interface subject to X’s terms of service and access policies. Interested researchers may contact the corresponding author for reasonable requests regarding the analytical approach or for additional details on the methodology.
